# Activation of peroxisome proliferator-activated receptor gamma is crucial for antitumoral effects of 6-iodolactone

**DOI:** 10.1186/s12943-015-0436-8

**Published:** 2015-09-17

**Authors:** Mario Nava-Villalba, Rosa E. Nuñez-Anita, Alexander Bontempo, Carmen Aceves

**Affiliations:** Instituto de Neurobiología, Universidad Nacional Autónoma de México-Juriquilla, Boulevard Juriquilla 3001, Juriquilla, Querétaro CP 76230 Mexico; Facultad de Medicina Veterinaria y Zootecnia, Universidad Michoacana de San Nicolás de Hidalgo, Morelia, Michoacán Mexico

**Keywords:** Iodine, Iodocompounds, PPARG, Arachidonic acid, Mammary cancer, MCF-7, Iodolactone

## Abstract

**Background:**

Molecular iodine (I_2_) exhibits antiproliferative and apoptotic effects on *in vivo* and *in vitro* cancer models. These effects are thought to be mediated by an iodinated arachidonic acid derivative, 6-iodolactone (6IL), and one of the proposed mechanisms is that 6IL activates Peroxisome Proliferator-Activated Receptors type gamma (PPARG). These receptors have been implicated in the inhibition of carcinogenic processes, in addition to their classical role in maintaining lipid and glucose homeostasis. The aim of this study was to determine whether PPARG participates in the 6IL antiproliferative and apoptotic effects on the mammary cancer cell line MCF-7.

**Methods:**

The 6IL/PPARG complex was inhibited by the PPARG antagonist GW9662, in both an endogenous and overexpressed (adenoviral vector infection) context, and stable PPARG-knockdown MCF-7 cells (RNA interference, confirmed with hydrolysis probes and Western blot), were used to corroborate the PPARG participation. 6IL effects on proliferation (measured by Trypan Blue exclusion) and apoptosis (phosphatidylserine identification by flow cytometer) were evaluated in conditions of chemical inhibition (GW9662) and silencing (RNA interference). A wound-healing assay was conducted on wild-type and stable PPARG-knockdown MCF-7 cells to evaluate the antimigrational effect of 6IL. Caspase-8 activity was evaluated to determine if the extrinsic pathway is involved in the effects of 6IL and I_2_ treatment.

**Results:**

Antiproliferative and pro-apoptotic 6IL effects require the activation of PPARG. In addition, wound-healing assays show that 6IL is able to inhibit MCF-7 cell migration and that PPARG plays a role in this phenomenon. Finally, the data exclude the participation of the extrinsic apoptotic pathway in 6IL- and I_2_-induced apoptosis.

**Conclusions:**

These results support the previously proposed mechanism, in which the I_2_ effects are mediated by 6IL, and they provide further support for the use of I_2_ as coadjuvant in breast cancer treatment.

## Background

6-Iodo-5-hydroxy-8,11,14-eicosatrienoic acid, δ-lactone (6-iodolactone or 6IL) is an iodinated arachidonic acid (AA) derivative and has been proposed to mediate the antitumoral effects of iodine [1–3]. Molecular iodine (I_2_), but not iodide (I^−^), exerts antineoplastic actions on diverse tissues (mammary, prostate and thyroid glands, and on melanoma, pancreas carcinoma and neuroblastoma cell lines), and various studies suggest that these effects can involve direct or indirect mechanisms [4–8]. In the direct effect, the oxidant/antioxidant property of I_2_ can disrupt the mitochondrial membrane potential and trigger mitochondrion-mediated apoptosis [6, 9]; on the other hand, the indirect path involves the generation of iodolipid intermediates, and there is evidence that 6IL could be one such intermediate. The presence of 6IL has been reported in normal and tumoral mammary gland from rats with continuous I_2_ supplements in their diet [2]. 6IL has also been observed in an *in vitro* model, in which an iodinated lipid co-migrating with the 6IL standard was detected in ^125^I_2_-treated MCF-7 cells [5].

At the molecular level, both I_2_ as 6IL generate antitumor effects through similar pathways. Both trigger a p53/p21-mediated cell growth arrest, and both induce Bax-caspases and AIF/PARP-1 apoptosis [1, 9]. In MCF-7 cells, 6IL supplement is accompanied by a significant stimulation of peroxisome proliferator-activated receptor type gamma (*PPARG*) and inhibition of PPAR alpha (*PPARA*) expression [10]. Moreover, using electrophoretic mobility shift assays (EMSAs), these authors demonstrated that 6IL is a PPAR ligand and can activate the PPAR response element (PPRE) (as shown by reporter gene transactivation assays). PPARs are involved in lipid metabolism, energy homeostasis and differentiation, and they have also been associated with positive or negative effects on carcinogenesis of various neoplasias [11–13]. While PPARA has been linked to carcinogenesis promotion [14, 15], PPARG or NR1C3 [16, 17] is proposed to be an antineoplastic agent, since it inhibits proliferation, induces apoptosis and promotes differentiation [18–22]. These opposing effects of PPARA and PPARG have been documented in normal and tumoral breast tissue and mammary cell lines [23, 24]. With respect to differentiation, 6IL induces cytoplasmic accumulation of lipid droplets, in a way comparable to that of the synthetic PPARG agonist rosiglitazone [10]. Thus, while previous work has shown a close relationship between PPARG and 6IL, here we use chemical and molecular approaches to demonstrate that the 6IL/PPARG complex is an intermediary in the antiproliferative and apoptotic effects of molecular iodine.

## Results

### GW9662 cancels the apoptotic and antiproliferative effects of 6IL

**Fig. 1:**
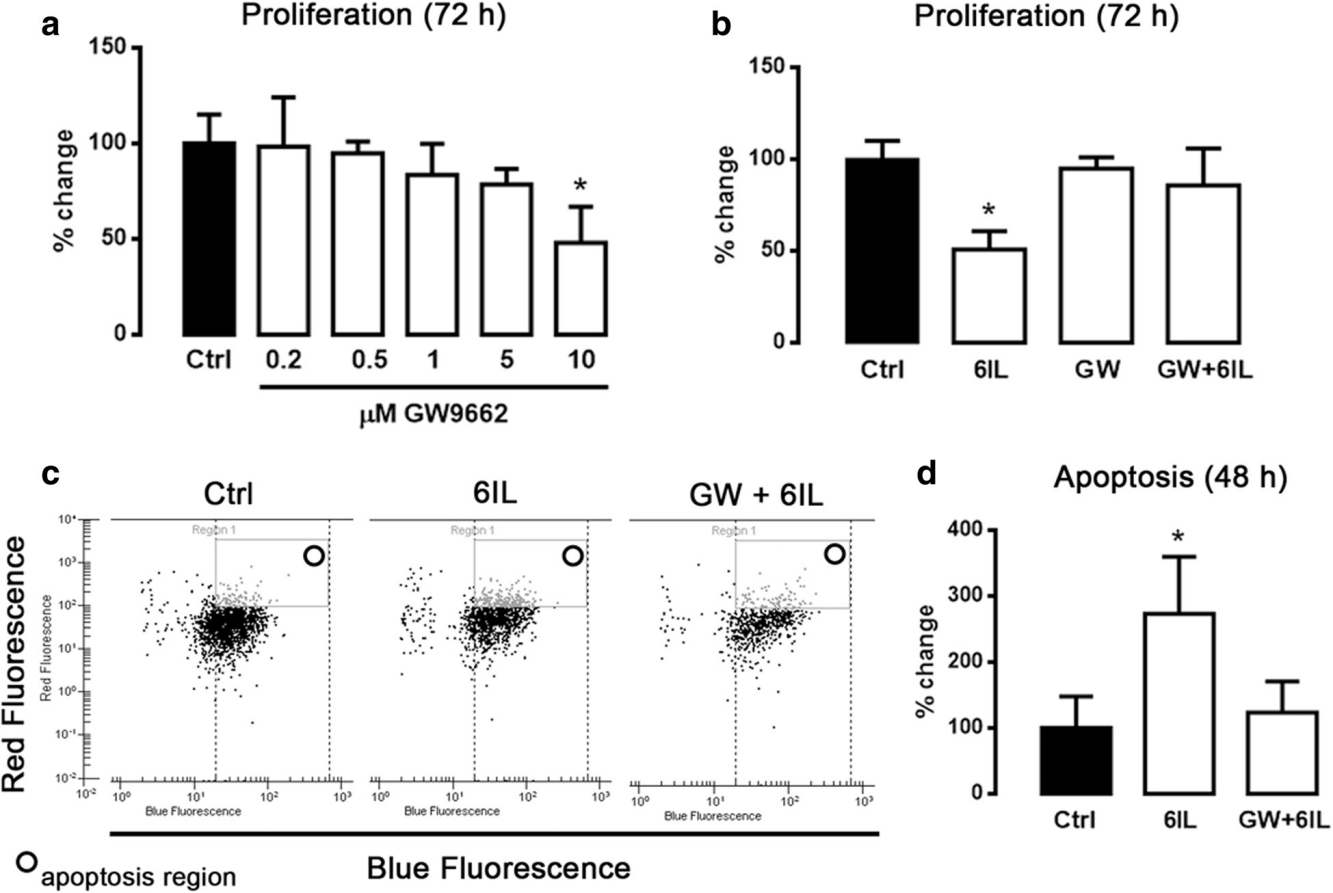
Antiproliferative and apoptotic effect of 6IL is blocked by GW9662 (GW). **a** MCF-7 cells were treated with increasing GW9662 concentrations to determine the optimal concentration that did not inhibit proliferation; 0.5 μM was selected for use in subsequent experiments. **b** Proliferation in the presence of 10 μM 6IL and after GW pretreatment was analyzed by the Trypan Blue exclusion assay. **c** Apoptosis was analyzed by flow cytometry (**c**, **d**). Results are expressed as percent change with respect to the control. Data are expressed as mean ± SD (*n* = 4 independent assays), and the asterisk indicates a significant difference with respect to the control (*P* < 0.05)

To demonstrate that PPARG activation plays a key role in the 6IL effects, we inhibited PPARG/6IL complex formation by blocking the PPARG ligand-binding domain with its highly specific antagonist GW9662. To determine the appropriate concentration of GW9662, i.e., a concentration that inhibited PPARG/6IL complex formation without causing the antiproliferative effects previously reported [25], we performed a dose–response curve (0.2 to 10 μM) for GW9662 in MCF-7 cells (Fig. 1a), and selected 0.5 μM for further experiments. Figure 1b shows that the 6IL supplement exerted a significant antiproliferative effect, which was prevented when the cells were pre-incubated (2 h) with GW9662. The 6IL treatment induced a significant, nearly two-fold increase in the apoptotic population compared to the control level (Fig. 1c-d), and this effect was blocked when the cells were pretreated with GW9662.

### Overexpression of PPARG enhances the antiproliferative and apoptotic effects of 6IL

**Fig. 2:**
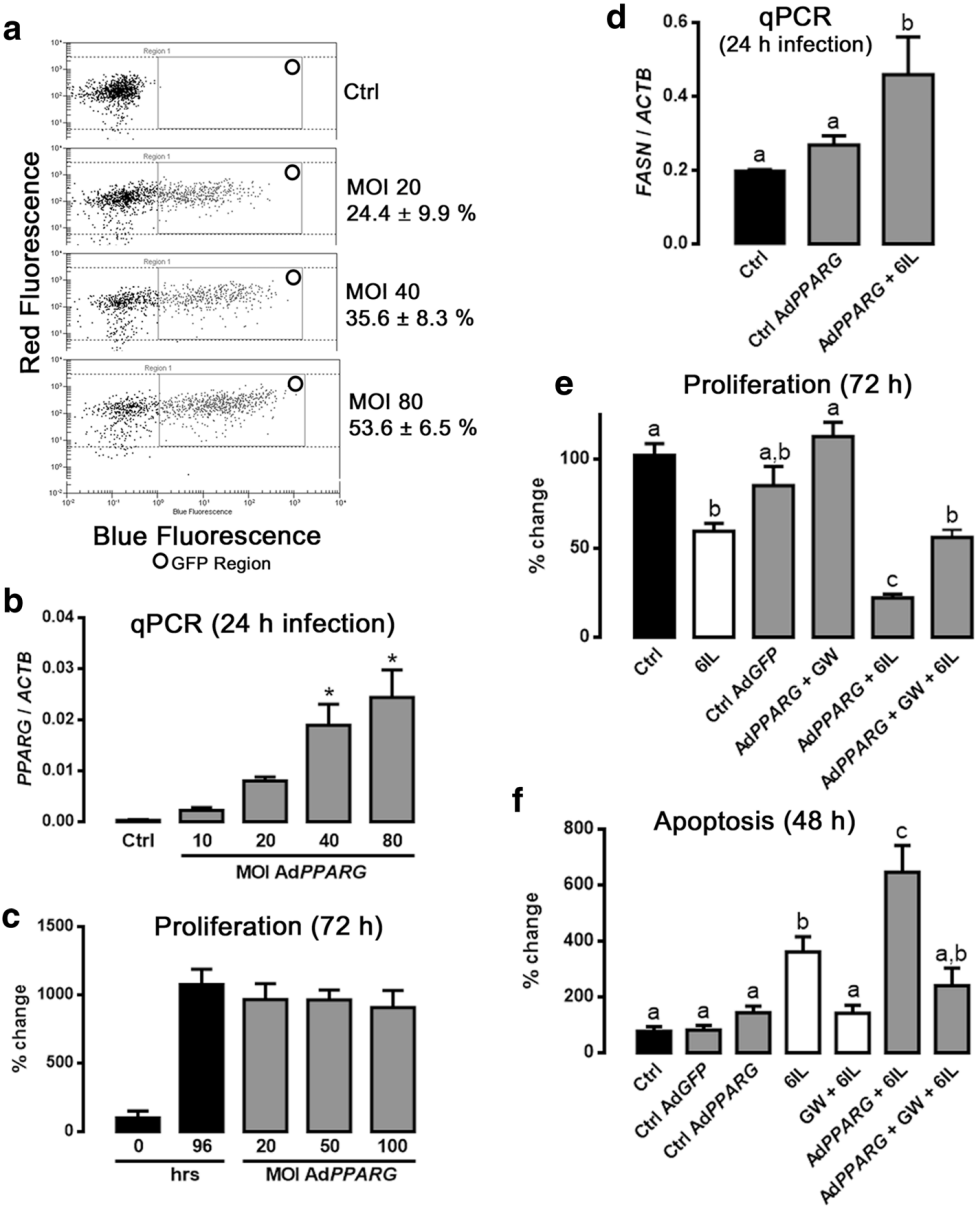
Overexpression of *PPARG* enhances antiproliferative and apoptotic effects of 6IL, and these enhancements are partially prevented by GW9662 (GW). **a** Representative panels of the infection-efficiency assay. MCF-7 cells were infected (24 h) with Ad*GFP* at increasing multiplicities of infection (MOIs). GFP expression is reported as a percentage of the number of events (%). **b** *PPARG* expression in Ad*PPARG*-infected cells was analyzed by qPCR and normalized to *ACTB* expression. **c** A range of MOIs was analyzed to determine the optimal level with no effect on proliferation; an MOI of 50 was selected for subsequent experiments. **d** Fatty acid synthase (*FASN*) expression was analyzed by the qPCR assay, and the results were normalized to *ACTB* expression. **e** Proliferation in the presence of 10 μM 6IL and after GW pretreatment was analyzed by the Trypan Blue exclusion assay. **f** The apoptotic effect of 6IL was analyzed by flow cytometry, and the results are expressed as percent change with respect to the control. Data are expressed as mean ± SD (*n* = 4 independent assays); the asterisks indicate significant differences with respect to the control (*P* < 0.05), and different letters indicate significant differences between groups (*P* < 0.05)

Figure 2a and b show the infection efficiency of MCF-7 with the *GFP*- and *PPARG*-adenoviral vectors, as measured by GFP intensity and mRNA amplification, respectively. Figure 2c shows the proliferation rate with ascending MOI (details in methods section), and a MOI of 50 was chosen for further experiments. Functional *PPARG* overexpression was confirmed by fatty acid synthase (*FASN*) expression, a PPARG-regulated gene (Fig. 2d). Figure 2e shows that the antiproliferative effect of the 6IL supplement was significantly intensified in the Ad*PPARG*-infected group compared to the uninfected group, and that preincubation with GW9662 only partially prevented this pronounced effect. In contrast, the overexpression of PPARG increased the sensitivity of these cells to the apoptotic effect of 6IL, and this effect was strongly inhibited by GW9662 (Fig. 2f).

### The silencing of PPARG expression blocks the antiproliferative and apoptotic effects of 6IL

**Fig. 3:**
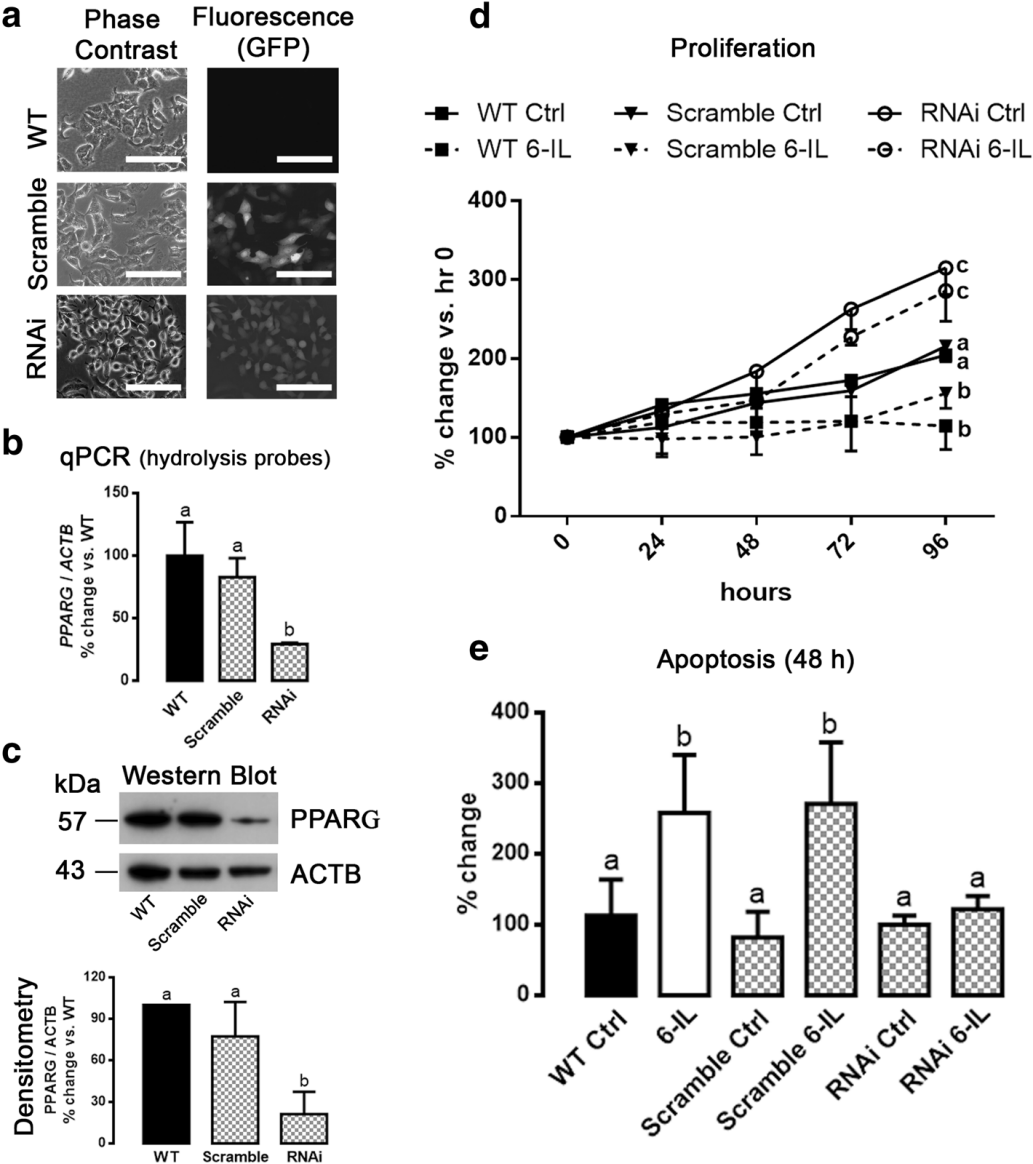
Knockdown of *PPARG* blocks the 6IL effects. MCF-7 cells were transfected with a retroviral silencing plasmid (HuSH™) containing a short, specific hairpin with RNA interference against *PPARG* (RNAi) or containing a scramble sequence (Scramble). **a** Representative microphotographs of GFP expression in stably transfected MCF-7 cells (20× objective, scale bar 50 μm). **b** *PPARG* expression was analyzed by qPCR and normalized to *ACTB* expression. **c** PPARG protein was quantified by Western blot and densitometry and is reported as percent change with respect to wild-type (WT) cells. **d** Proliferation in the presence of 10 μM 6IL was analyzed by Trypan Blue exclusion. **e** The apoptotic effect of 6IL was analyzed by flow cytometry, and the results are expressed as percent change with respect to the corresponding control. Data are expressed as mean ± SD (*n* = 4 independent assays), and different letters indicate significant differences between groups (*P* < 0.05)

To confirm the stable transfection of RNAi against *PPARG* mRNA in selected cells, GFP expression (from the *GFP* gene co-inserted with the RNAi into the HuSH™ plasmid) was assessed (Fig. 3a). *PPARG* silencing was confirmed by qPCR with specific Hydrolysis Probes (Fig. 3b and Table 1) and Western blot (Fig. 3c). Wild type (WT), Scramble-*PPARG* (Scramble) and RNAi-*PPARG* (RNAi) MCF-7 cells were treated with 10 μM 6IL over a 96-h period, and their proliferation rates were evaluated. Figure 3d shows that WT and Scramble-*PPARG* MCF-7 cells had the same proliferation rates, whereas RNAi-*PPARG* MCF-7 cells exhibited significantly faster proliferation. The 6IL supplement inhibited proliferation of WT and Scramble-*PPARG* MCF-7 cells, whereas it had no effect on the *PPARG*-knockdown group (RNAi-*PPARG*). Figure 3e summarizes the apoptotic effect of 6IL in these groups, showing significant apoptosis on WT and Scramble-*PPARG* cells and no effect on RNAi-*PPARG* MCF-7 cells.

**Table 1:** Real-time PCR primer sequences

Gene	Sequence (5′–3′)		Annealing temp (°C)	ID
*FASN*	Sense	GGAATGGGAAGACACCTATGGA	62	GenBank NM_004104.4
	Antisense	AGAGAGAGCTCAGATACGTTGAC		
*PPARG*	Sense	TCTCTCCGTAATGGAAGACC	62	GenBank L40904.2
	Antisense	GCATTATGAGACATCCCCAC		
*ACTB*	Sense	CCATCATGAAGTGTGACGTTG	62	GenBank NM_001101.3
	Antisense	ACAGAGTACTTGCGCTCAGGA		
*PPARG* Hydrolysis Probe	Sense	GATGTCTCATAATGCCATCAGGTT	60	RTPrimerDB ID: 5471
	Antisense	GGATTCAGCTGGTCGATATCACT		
	Probe	CCAACAGCTTCTCCTTCTCGGCCTG		
*ACTB* Hydrolysis Probe	Sense	TCCTTCCTGGGCATGGAG	60	RTPrimerDB ID: 2418
	Antisense	AGGAGGAGCAATGATCTTGATCTT		
	Probe	CCTGTGGCATCCACGAAACTACCTTC		

### 6IL inhibits MCF-7 cell migration, but this effect is blocked by silencing PPARG

**Fig. 4:**
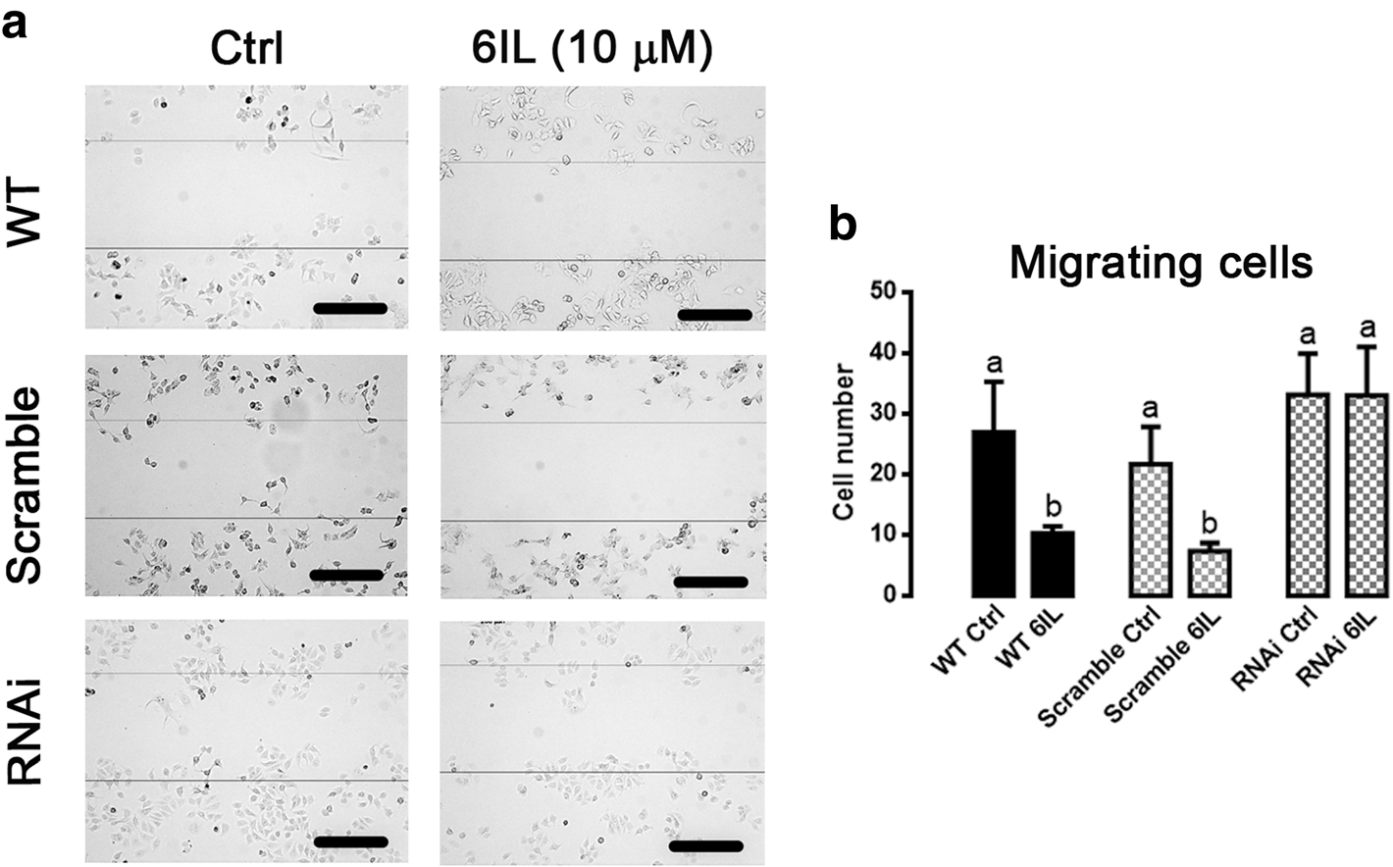
6IL inhibits MCF-7 migration and this effect is partially reverted by *PPARG* knockdown. Wild type, Scramble- and RNAi-*PPARG* transfected MCF-7 cells were treated with 10 μM 6IL for 24 h, and the number of cells that had migrated into the scratch during this time was determined (10× objective, scale bar 200 μm, average scratch width 377 μm). **a** Representative microphotographs of wound-healing assays. **b** Migrating cells (total number). Data are expressed as mean ± SD (*n* = 3 independent assays), two-way ANOVA and Tukey’s multiple comparison tests were performed; different letters indicate significant differences between groups (*P* < 0.05)

Groups were treated for 24 h with 10 μM 6IL or vehicle, and the migration rate was evaluated by a wound-healing assay (Fig. 4a). WT and Scramble-*PPARG* MCF-7 cells treated with 6IL showed significant inhibition of migration, around 60 %; however, 6IL did not change the migration rate of RNAi-*PPARG* MCF-7 cells (Fig. 4b).

### The 6IL induction of apoptosis is not activated by the extrinsic pathway

**Fig. 5:**
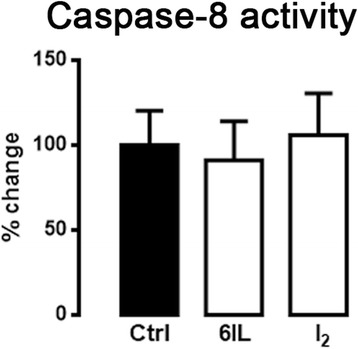
Extrinsic apoptotic pathway is not involved in the induction of apoptosis by 6IL or I_2_. MCF-7 cells were treated with 10 μM 6IL or 100 μM I_2_ for 48 h. Caspase-8 activity was analyzed by a colorimetric assay and normalized for the amount of protein. The results are reported as percent change with respect to the control. Data are expressed as mean ± SD (*n* = 3 independent assays). There were no significant differences between groups

To evaluate the participation of the extrinsic pathway in the 6IL or I_2_ effects on apoptosis, caspase-8 activity was evaluated. Neither 10 μM 6IL nor 200 μM I_2_ treatments produced any change in caspase-8 activity (Fig. 5).

## Discussion

A significant body of data from *in vivo* experiments [4, 26–29], clinical trials [30–33], and epidemiological reviews [34–36] support the notion that molecular iodine has a protective effect against the progression of neoplastic diseases and inflammatory/proliferative pathologies. An important fact to consider is that these beneficial effects occur only at relatively high iodine concentrations (milligrams/day) and only in those tissues that are able to capture iodine. In the case of mammary and prostate glands, both normal and tumoral cells internalize I_2_ by a sodium/iodine symporter (NIS)-independent mechanism [1, 4, 5, 8, 37] that seems to involve a facilitated diffusion process (saturable and dependent on protein synthesis) [5]. It has not yet been determined how I_2_ reacts with cell components, but I_2_ is known to bind covalently to proteins and lipids [5]. The iodination of fatty acids generates several derivatives, but those from arachidonic acid (AA), like 6IL, are especially relevant since endogenous 6IL has been detected [38, 39], and it generates a range of biological effects [3]. The proposal that PPARG plays a role in the antitumor effects exhibited by I_2_ after its conversion to 6IL is supported by the following evidence: 1) 6IL can be identified in both *in vitro* and *in vivo* models after administration of I_2_ [1, 2]; 2) expression of *PPARG* is increased in I_2_- or 6IL-supplemented conditions (possibly through an autoregulation mechanism) [2, 10]; 3) the antiproliferative effect of I_2_ is only observed in cancer cells, which contain elevated concentrations of AA [2]; and 4) when incubated with total protein, 6IL can activate the PPAR response element (shown by luciferase transactivation assays) and is capable of inducing MCF-7 cells to accumulate lipid droplets in the same manner as the highly specific agonist rosiglitazone [10]. Previously, our group showed that the molecular antiproliferative/apoptotic actions of the 6IL/PPARG complex include the activation of p53 and the consequent increase of p21 expression, thereby inducing cell arrest and Bax-caspase expression, which activates the intrinsic apoptosis pathway [1, 28, 29]. GW9662 irreversibly modifies Cys^285^ in the PPARG ligand-binding domain with an IC_50_ in the nanomolar range [40], as compared to the micromolar IC_50_ of 6IL [1, 10]. This very high affinity can explain the almost complete inhibition by GW9662 observed in control cells. However, proliferation was not totally blocked in cells overexpressing *PPARG*. By its nature, the adenoviral system forces the cell to maintain unregulated expression; therefore, it is possible that the GW9662 concentration was not sufficient to saturate the PPARGs due either to their overexpression from the vector or to well-established, positive, autoregulatory mechanisms that maintain a high and continuous expression of *PPARG* [41, 42]. In contrast, the 6IL-induced effect on apoptosis is totally blocked by GW9662 in both control and *PPARG*-overexpressing cells. Thus, partial PPARG blockage may unmask this dual action of 6IL, in which only a few free PPARG receptors are required for the antiproliferative effect (maybe cell cycle arrest), but additional available receptors are needed to induce apoptosis. Similar dose-dependent responses of PPARG ligands have been described in the literature previously, showing induction of arrest at low concentrations, whereas at high levels they trigger apoptosis [43, 44]. Moreover and although molecular mechanisms for these preferential responses have not been explored, differential recruitment of coactivator cannot be excluded. More than 300 cofactors (coactivators, coregulators, corepressors, etc.) have been identified for the nuclear receptor family. It has been shown that these elements interact with one another and the same ligand modify transcription of many genes [45]. Interestingly, in the *PPARG*-overexpressing cells, 6IL administration also induces increased expression of *FASN*, a PPARG-regulated gene that is related to differentiation [46, 47]. This observation is consistent with the differentiation effect of 6IL, observed as lipid accumulation in MCF-7 cells [10].

In relation with silencing the expression of PPARG by molecular tools, our results show that the proliferation rate of RNAi-*PPARG* MCF-7 cells was two-fold greater than that of MCF-7 control cells, corroborating similar reports in other cancer cells [48]. The findings that MCF-7 cells with *PPARG* knockdown are insensitive to both the antiproliferative/apoptotic as well as to the anti-migratory effects of 6IL are clear indications that these receptors have an important role in the antineoplastic actions of I_2_ or 6IL. The next step is to determine if 6IL affects the cell’s invasive characteristics, since the activity of some metalloproteinases and other invasive markers has been blocked by PPARG ligands in several cancers [49, 50].

Finally, it has been described that I_2_ or 6IL induces apoptosis effects through at least two pathways: AIF/PARP1 and Bax-caspases [1, 9]; Bonofiglio *et al*. [51] reports that PPARG is able to activate the promoter of the *FasL* gene, thereby inducing extrinsic apoptosis in MCF-7 cells; this observation was reinforced recently by similar findings in human colorectal cancer cells [52]. In the present work we explored if the extrinsic apoptotic pathway is activated by the 6IL/PPARG complex. Our results show that caspase-8 activity is not increased by I_2_ or by 6IL treatment; therefore, it seems that the apoptotic effects of I_2_ and 6IL are mediated exclusively by intrinsic apoptosis pathways.

## Conclusion

In summary, our data demonstrate that the antitumor effects of 6IL are mediated by PPARG; moreover, the induction of *FASN* expression strengthens previous observations that 6IL also exerts differentiating effects (adipogenetic induction [10]). 6IL inhibits migration, an effect that may involve PPARG activation. Additionally, 6IL-induced apoptosis does not involve an extrinsic apoptotic pathway. Together, these data reinforce the notion that 6IL is a mediator of I_2_ effects, and it strengthens the proposal that I_2_ can be a useful coadjuvant in treatment of cancer in tissues that take up iodine.

## Methods

### Materials and cell culture

Dulbecco’s modified Eagle’s medium (DMEM), fetal bovine serum (FBS), penicillin/streptomycin, puromycin and trypsin solution were supplied by GIBCO (Grand Island, NY, USA). Arachidonic acid (AA) (purity >99 %) was used to synthesize 6IL was from Calbiochem (La Jolla, CA, USA). GW9662 (purity >98 %), a specific PPARG antagonist, was obtained from Sigma-Aldrich (St. Louis, MO, USA). All other chemicals were of the highest grade of purity commercially available. The breast cancer cell line MCF-7 was obtained from ATCC®. The cells were maintained in Basal Medium (DMEM supplemented with 10 % FBS, 100 U/mL penicillin, 100 μg/mL streptomycin) at 37 °C and 5 % CO_2_ prior to experiments.

### Chemical synthesis of 6IL

6IL was synthesized with a modification of the method of Monteagudo [53]. Briefly, to a solution of AA (65 mg) in acetonitrile (0.8 mL) was added a solution of iodine (156 mg) in acetonitrile (8 mL) at 4 °C. The solution was kept under N_2_, stirred for 4 h at room temperature and protected from light. The solution containing crude product was concentrated under low nitrogen flow to 0.5 ml and separated on preparative TLC using the solvent system CH_2_Cl_2_/MeOH (97.5:2.5). The reaction products, AA (rf: 0.5) and 6IL (rf: 0.88) standards were visualized by iodine vapors, and the 6IL synthesized was eluted with the same solvent and concentrated under low nitrogen flow.

### Adenoviral infection

Recombinant adenoviruses expressing human *PPARG* (Ad*PPARG*) were provided by Dr. Steals (Institut Pasteur de Lille, France). Adenovirus expressing *GFP* (Ad*GFP*), provided by Dr. Hernandez-Gutiérrez (Universidad Panamericana, México), was used to confirm infection and as control. Viruses were multiplied in HEK-293 cells (provided by Dr. Martínez-Torres, Instituto de Neurobiología UNAM, México). Adenoviral titer was determined by the MOI (multiplicity of infection) test (AdEasy™ Vector System, application manual version 1.4). Prior to treatment with GW9662 and/or 6IL, MCF-7 cells were infected at an MOI of 50 for 24 h in basal medium.

### Stable transfection with shRNA against PPARG (RNAi-PPARG)

Retroviral silencing plasmid (HuSH™) containing a short, specific hairpin with RNA interference for *PPARG*, a puromycin resistance gene, a *tGFP* gene and a U6 promoter was obtained from Origen Technologies, Inc. (Rockville, MD, USA) (RNAi-*PPARG* cells). A similar plasmid but with a scrambled sequence of the short RNAi hairpin against *PPARG* (Scramble-*PPARG* cells) was used as internal control. MCF-7 cells were transfected with 5 μg of plasmid and Xfect™ Transfection Reagent (Clontech Laboratories, Mountain View, CA, USA) according to the manufacturer’s instructions, and they were maintained in culture for 48 h; after this time the transfected cells were selected with 1.0 μg/mL puromycin daily over 2 weeks and expanded. Transfection of the cells was confirmed by *tGFP* expression using an IX50 Olympus microscope with inverted reflected light fluorescence system (Olympus Inc., NY, USA), or by an Agilent 2100 bioanalyzer using an On-Chip flow cytometry system (Agilent Technologies, USA). Knock-down of *PPARG* expression was evaluated by quantitative real time PCR (qPCR) with the specific Hydrolysis Probes (Sigma-Aldrich Co., MO) shown in Table 1 and by Western Blot. The selected and stably transfected population was maintained under “light pressure” (0.5 μg/mL puromycin) over the course of the experiments.

### Experimental groups

The experiments were carried out on Ad*PPARG*-MCF-7-infected cells, on Scrambled- or RNAi-*PPARG*-MCF-7-transfected cells and on wild-type MCF-7 cells. The cells were cultured at a density of 5 × 10^5^ cells in 60-mm culture dishes (Nalge Nunc International, Naperville, IL) for qPCR assays; 4 × 10^4^ cells/well in 24-well culture plates for proliferation assays, 2 × 10^5^ cells in 60-mm culture dishes for apoptosis experiments, 3 × 10^6^ cells in 100-mm culture dishes for Western blot assays, 8 × 10^5^ cells in 60-mm culture dishes for wound-healing assays, and 5 × 10^6^ cells in 100-mm culture dishes for caspase-8 activity assays. All cells were seeded in complete basal medium and incubated for 24 h before treatment; at the beginning of experiments the medium was changed to low serum (0.5 %)-containing medium. The cells were treated for 24, 48, 72 or 96 h, (depending on the experiment) at 37 °C with 10 μM 6IL, 0.5 μM GW9662 or vehicle (ethanol) diluted before application in low serum-containing medium. In the GW9662/6IL-treated group, GW9662 was administered 2 h prior to 6IL treatment.

### Quantitative PCR

Total RNA was extracted from cells using TRIzol® reagent (Life Technologies, Thermo Fischer Scientific, MA, USA) according to the manufacturer’s instructions. Two micrograms of total RNA was reversed transcribed using the Superscript II system (Invitrogen, Carlsbad, CA). qPCR was performed on the sequence detector system Roto-Gene 3000 (Corbett Research, Mortlake, Australia) using SYBR green as a marker for DNA amplification. The reaction was performed with 1 μL cDNA template and the Maxima SYBR Green/ROX qPCR Master Mix (Thermo Fischer Scientific, MA), using 40 cycles of three-step amplification (94 °C for 30 s, 55–62 °C for 30 s, 72 ° C for 30 s) and the gene-specific primers listed in Table 1. PCR generated only the expected specific amplicon, which was demonstrated in each case by the melting temperature profile (dissociation curve) and by electrophoresis of 5 μL of the PCR product through a 2 % agarose gel in TAE buffer then visualization by ethidium bromide. No PCR products were observed in the absence of template. Gene expression was calculated using the D cycle threshold method and normalized to the content of *ACTB*, a non-regulated housekeeping gene. All measurements were performed in triplicate.

### Western blot

Pellets of whole protein were obtained from lysed cells using RIPA buffer with complete Mini, EDTA-free protease inhibitor cocktail tablets (Roche Diagnostics GmbH, Germany). The extracted proteins were quantified by the Bradford method (Bio-Rad protein assay; Hercules, CA) and analyzed by Western blot. SDS polyacrylamide gel electrophoresis was performed as described [10], using stacking and resolving gels (the latter with 15 % acrylamide). Samples containing 50 μg of protein were applied to each lane. After electrophoresis, the proteins were electrotransferred to a nitrocellulose membrane (Bio-Rad, Germany), which was then blocked overnight with TBS containing 5 % non-fat dry milk. The membranes were rinsed and treated with polyclonal anti-PPARG antibody (sc-7196, 57 kDa, 1:1000, Santa Cruz Biotechnology Inc., USA) and anti-ACTB (sc-1616, 43 kDa, 1:10,000, Santa Cruz Biotechnology Inc.) as a protein-loading control. Protein bands were visualized using a chemiluminescent detection system (ECL, Amersham Biosciences, UK). Densitometry was performed with Image Lab™ software (Bio-Rad Laboratories, CA, USA), and chemiluminescence was normalized to the level of ACTB protein.

### Proliferation assays

The effects on proliferation of 10 μM 6IL, 0.5 μM GW9662 and the GW9662/6IL mixture were analyzed using the Trypan Blue dye-exclusion assay. The cells (4 × 10^4^/well) were cultured in DMEM supplemented with 10 % FBS on 24-well culture plates. Control cells were treated with 0.1 % ethanol (solvent for 6IL). After a 72-h treatment, the cells were harvested with trypsin-EDTA 0.05 % (Life Technologies Brand of Thermo Fischer Scientific, MA, USA). Trypan Blue (0.4 %) was added; those cells that excluded the dye were counted with a hemocytometer under the microscope (Leica Inverted Microscope DMIL model, Germany); proliferation is reported as fold change with respect to control.

### Apoptosis assays

Apoptosis was assessed by detecting phosphatidylserine on the external surface of the cell membrane with annexin V conjugated to the Cy5 fluorophore. To mark cells with intact cell membranes, Calcein-AM was used. Apoptosis was assessed by an Agilent Bioanalyzer 2100 and apoptosis kit following the manufacturer’s instructions (Agilent Technologies, USA). Briefly, after treatment the cell density was adjusted to 1 × 10^6^ per mL in 200 μL basal medium, and 200,000 cells were transferred to a 1.5-mL microtube and centrifuged at 2500 rpm for 3 min; the supernatant was discarded. The pellet was resuspended in 200 μL of 1X Binding Buffer (provided by the manufacturer), and 1 μL of Annexin V (Abcam®, UK) was added and incubated at room temperature for 10 min; after centrifugation at 2500 rpm for 3 min the supernatant was removed, and 1 μL each of Calcein-AM and Fluorolink Cy5 in 200 μL of Binding Buffer were added to the pellet and incubated for 10 min. Finally, the cells were collected by centrifugation and resuspended in Cell Buffer (provided by the manufacturer). A 10-μL aliquot of cells from each treatment was placed on the cell fluorescence LabChip® together the solutions provided in the kit. The apoptotic cells are reported as a percentage of the number of events; this population is delimited in a standardized region by the program Cell-Chip Agilent 2100 Expert software (Agilent Technologies, USA).

### Wound-healing assay

RNAi-*PPARG* MCF-7 cells, Scramble-*PPARG* MCF-7 cells and wild-type MCF-7 cells, 8 × 10^5^ in each case, were seeded on 60-mm culture plates and incubated at 37 °C and 5 % CO_2_. After 24 h, the attached cells were scratched three times in parallel with a 1000-μl pipette tip (~377 μm) and treated with 10 μM 6IL or vehicle (ethanol) for 24 h. After this time, three new parallel wounds were performed as width control; the number of migrating cells was obtained by counting the number of cells inside this width control. The count was measured by Leica Application Suite Version 2.8.1., and the images were captured with a 10× objective lens using a Leica DM 2500 microscope and DFC 420 camera (Leica Microsystems, CMS GmbH, Germany). The results are presented as number of migrating cells of treated (6IL) vs. control (Ctrl) groups (mean ± SD). At least three fields were analyzed for each plate, and each group was analyzed in three independent experiments.

### Caspase-8 activity assay

The caspase-8/FLICE activity was measured using a commercial kit (ApoTarget, Invitrogen Co., CA, USA) according to manufacturer’s instructions. Briefly, 5 × 10^6^ MCF-7 cells were collected after a 48-h incubation with I_2_ or 6IL. The cell pellet was lysed, and an aliquot containing 200 μg of protein was incubated with DTT (10 mM) and a colorimetric substrate, IETD-*p*-nitroanilide; (*p*NA, final concentration 200 μM) at 37 °C for 120 min in the dark. The samples were then read in the spectrophotometer at 405 nm. The absorbance of *p*NA from a treated sample was compared with an untreated control to determine the fold increase in caspase-8 activity.

### Statistical analysis

One- or two-way ANOVA was performed to determine the significance of differences between groups, followed by Tukey’s test for the significance of differences among multiple experimental groups. Data are expressed as mean ± standard deviation (SD), and values with *P* < 0.05 were considered statistically significant. In the figures, an asterisk indicates significant differences with respect to the control (*P* < 0.05), and different letters indicate significant differences between groups (*P* < 0.05).
